# Increased mitochondrial coupling and anaerobic capacity minimizes aerobic costs of trout in the sea

**DOI:** 10.1038/srep45778

**Published:** 2017-03-31

**Authors:** Jeroen Brijs, Erik Sandblom, Henrik Sundh, Albin Gräns, James Hinchcliffe, Andreas Ekström, Kristina Sundell, Catharina Olsson, Michael Axelsson, Nicolas Pichaud

**Affiliations:** 1 Department of Biological and Environmental Sciences, University of Gothenburg, Gothenburg, Sweden; 2 Department of Animal Environment and Health, Swedish University of Agricultural Sciences, Skara, Sweden; 3 Département de chimie et biochimie, Université de Moncton, Moncton, NB, Canada

## Abstract

Anadromy is a distinctive life-history strategy in fishes that has evolved independently many times. In an evolutionary context, the benefits of anadromy for a species or population must outweigh the costs and risks associated with the habitat switch. The migration of fish across the freshwater-ocean boundary coincides with potentially energetically costly osmoregulatory modifications occurring at numerous levels of biological organization. By integrating whole animal and sub-cellular metabolic measurements, this study presents significant findings demonstrating how an anadromous salmonid (*i.e.* rainbow trout, *Oncorhynchus mykiss*) is able to transform from a hyper- to hypo-osmoregulatory state without incurring significant increases in whole animal oxygen consumption rate. Instead, underlying metabolic mechanisms that fuel the osmoregulatory machinery at the organ level (*i.e.* intestine) are modulated, as mitochondrial coupling and anaerobic metabolism are increased to satisfy the elevated energetic demands. This may have positive implications for the relative fitness of the migrating individual, as aerobic capacity may be maintained for locomotion (*i.e.* foraging and predator avoidance) and growth. Furthermore, the ability to modulate mitochondrial metabolism in order to maintain osmotic balance suggests that mitochondria of anadromous fish may have been a key target for natural selection, driving species adaptations to different aquatic environments.

The migration of fish across the freshwater-ocean boundary, *i.e.* anadromy, is widely considered to provide considerable adaptive and selective advantages in temperate latitudes, as the oceans can provide richer food resources in comparison to freshwater environments^1^,^2^,^3^,^4^. For example, various species within the Salmonidae family (a family regarded as ‘icons’ for the anadromous life-history strategy^3^) experience substantial increases in their daily growth rate when at sea (*e.g.* up to 50% increase in Chinook salmon, *Oncorhynchus tshawytscha*^5^), which allows them to grow larger than non-migratory conspecifics^1^,^6^,^7^. The larger body size of anadromous individuals incur selective advantages such as increased fecundity, larger eggs and an intraspecific competitive advantage with respect to occupying spawning grounds and competing for mates^1^,^3^. However, life at sea coincides with a wide range of potentially energetically costly, osmoregulatory modifications occurring at numerous levels of biological organization (*i.e.* whole-animal to sub-cellular level)^4^,^8^.

To maintain osmotic homeostasis, anadromous salmonids such as rainbow trout (*Oncorhynchus mykiss*) migrating across the freshwater-ocean boundary initiate an immediate drinking response^9^, which triggers further physiological changes^10^. Specifically, imbibed seawater undergoes substantial oesophageal desalination before entering the intestine, whereupon further absorption of monovalent ions via a range of co-transporters, exchangers, ion channels and Na^+^/K^+^-ATPase (NKA) drive intestinal water absorption^10^,^11^,^12^. The excess monovalent ions absorbed by the gastrointestinal tract, as well as the additional salt gain occurring across all epithelial surfaces, are then branchially excreted via mitochondria-rich cells^13^. Long-term salinity tolerance and hypo-osmoregulatory ability of salmonids has been demonstrated to involve a wide raft of permanent regulatory modifications that result in increased levels of branchial and intestinal NKA activity, intestinal fluid absorption, luminal precipitation of divalent cations, as well as an elevated cardiac output and gastrointestinal blood flow^9^,^10^,^14^,^15^,^16^,^17^.

Osmoregulation in marine environments is a metabolically demanding process (*e.g.* elevated ATP demand of branchial and intestinal NKA) and maintaining osmotic homeostasis in the sea has the potential to be more energetically costly than in freshwater habitats (as might be expected from the NaCl gradient between *milieu intérieur* and external medium^18^). Yet, there seems to be no universal trend with respect to metabolic cost in relation to environmental salinity, as estimates of osmoregulatory costs in euryhaline teleosts (primarily based on whole animal oxygen consumption measurements, 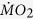) vary considerably from a few percent up to one-third of an animal’s metabolic rate^8^. However, a recent review^19^ highlighted that 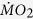 may only be a partial proxy for energy metabolism as the amount of ATP generated per unit of oxygen consumed can vary significantly^20^, within and between individuals, as well as between populations and environments (see ref. 19 and references within). Therefore, when investigating the link between energy metabolism and animal performance, 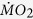 should preferably be used in combination with measures of mitochondrial metabolism^19^.

Few studies have estimated the energetic cost of osmoregulation at the organ level (*e.g.* gills and kidney) and suggest that these costs may only contribute towards a minor proportion of an animal’s metabolic rate^21^,^22^,^23^,^24^,^25^. Interestingly, relatively little is known about the energetic demands of the intestine where active, ATP demanding transport mechanisms are essential for osmoregulation in seawater^10^,^12^. Recent studies have shown that gastrointestinal blood flow in rainbow trout (~30% of cardiac output in freshwater) doubles after acclimation to seawater, as cardiac output is elevated and an increasing proportion of blood is directed towards the gastrointestinal system^9^,^16^,^17^. Thus, assuming that the intestine has a similar oxygen extraction as other tissues, it could be speculated that the metabolic cost of intestinal processes in seawater represents a sizeable portion of the animal’s metabolic rate. Moreover, one could also expect that the increased demand for ATP by the intestinal NKA of salmonids in seawater would impact the dynamics of mitochondrial metabolism, which produces the majority of cellular ATP. However, the underlying mechanisms for adjusting mitochondrial respiration in the intestine of an anadromous salmonid have to our knowledge never been investigated, nor have the relative contributions of aerobic and/or anaerobic metabolism in this organ been studied.

Therefore, the present study integrates whole animal and sub-cellular measurements of metabolism to characterize and understand the energy metabolism of an anadromous salmonid (*i.e.* rainbow trout) crossing the freshwater-ocean boundary, as well as providing insight into the life-history trade-offs associated with anadromy. We hypothesized that the return of osmotic balance in rainbow trout following a transfer to seawater would coincide with an increased intestinal NKA activity. The elevated ATP demand by intestinal NKA would consequently result in an elevated intestinal and whole animal metabolism. To test this hypothesis, we investigated the temporal dynamics of osmotic balance (*i.e.* plasma osmolality and ion concentrations), intestinal NKA activity, 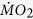, intestinal mitochondrial respiration rates, as well as enzymatic activities of citrate synthase (CS, an enzyme of the tricarboxylic acid cycle and a marker of aerobic capacity) and lactate dehydrogenase (LDH, a marker of anaerobic capacity) during a 35-day seawater acclimation period.

## Results

### Plasma osmolality and intestinal NKA activity

**Figure 1:**
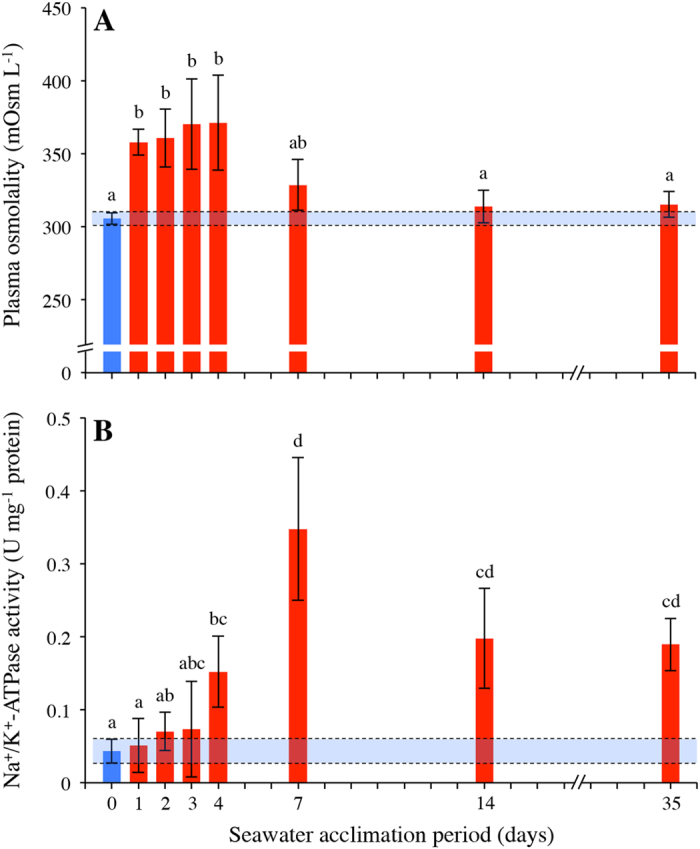
Osmotic status and intestinal osmoregulatory activity of rainbow trout in freshwater and seawater. (**A**) plasma osmolality and (**B**) intestinal NKA activity of rainbow trout in freshwater (blue bar) and after a transfer and/or subsequent acclimation to seawater (red bars) at 10.0 ± 0.1 °C (n = 8 individual trout for each time point). Variables are reported as means and the error bars represent the upper and lower bands of the 95% CI for each mean (horizontal blue shaded rectangle represents 95% CI for freshwater acclimated trout). Dissimilar letters represent significant differences among groups (*p* < 0.05, Welch’s ANOVA, Games-Howell post-hoc analysis).

Plasma osmolality was significantly affected by the transfer and/or subsequent acclimation to seawater (Welch’s ANOVA F_7,22.956_ = 27.642, *p* < 0.001, Fig. 1A). Plasma osmolality increased from ~300 mOsm L^−1^ in freshwater to 360–370 mOsm L^−1^ during the first 4 days in seawater (*p* ≤ 0.001 for comparisons between freshwater and day 1–4 of seawater acclimation). After 7 days of seawater acclimation, plasma osmolality gradually returned to, and was maintained at, a level that was not different to that observed in freshwater (*p* = 0.161, 0.717, 0.371 for comparisons between freshwater and day 7, 14, 35, respectively). Plasma osmolality was positively correlated with plasma [Na^+^] (r_(62)_ = 0.89, *p* < 0.001), [K^+^] (r_(61)_ = 0.46, *p* < 0.001) and [Ca^2+^] (r_(62)_ = 0.54, *p* < 0.001).

NKA activity was also significantly affected by the transfer and/or subsequent acclimation to seawater (Welch’s ANOVA F_7,23.337_ = 18.847, *p* < 0.001, Fig. 1B). After 4 days of seawater acclimation, intestinal NKA activity was higher than that observed in freshwater-acclimated rainbow trout (*p* = 0.012), and at day 7 it reached a maximum value of 0.35 ± 0.10 U mg^−1^ protein before gradually returning to, and stabilizing at, an activity level of ~0.2 U mg^−1^ protein. After 35 days of seawater acclimation, intestinal NKA activity remained ~5-fold higher than that of freshwater acclimated individuals (*p* < 0.001).

### Metabolic implications of seawater acclimation

**Figure 2:**
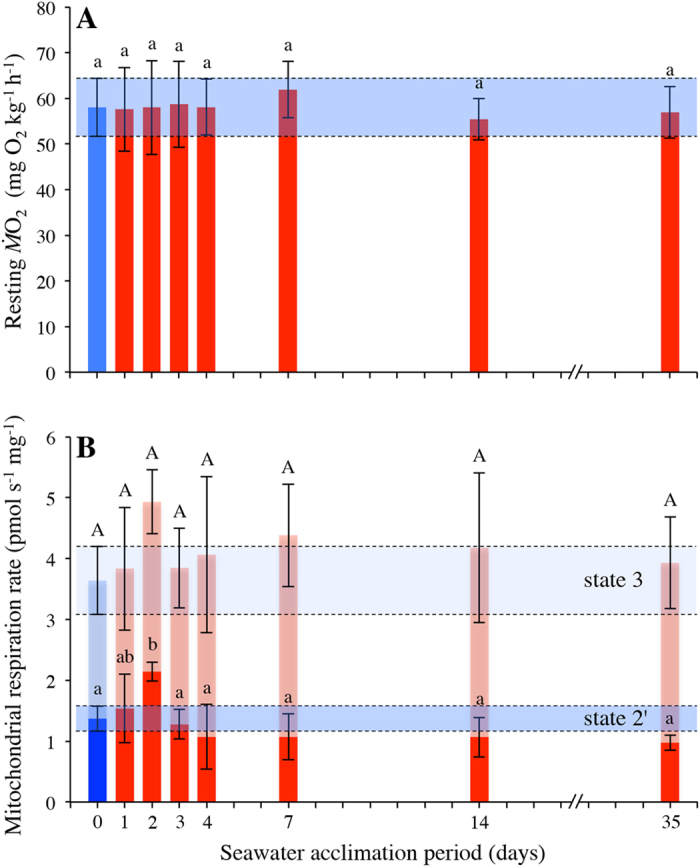
Whole animal and sub-cellular metabolic costs of rainbow trout in freshwater and seawater. (**A**) resting whole animal oxygen consumption (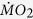), (**B**) state 2′ (solid bars in foreground) and state 3 intestinal mitochondrial respiration rates (semi-transparent bars in background) of rainbow trout in freshwater (blue bar) and during the transfer and subsequent acclimation to seawater (red bars) seawater acclimation at 10.0 ± 0.1 °C. Sample sizes for 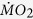 (n = 8 individual trout for each time point) and intestinal mitochondrial respiration measurements (n = 7 individual trout for each time point except day 3 and 35 where n = 8) were different. Variables are reported as means and the error bars represent the upper and lower bands of the 95% CI for each mean (horizontal blue shaded rectangles represents 95% CI for freshwater acclimated trout). Dissimilar lower case (state 2′) and upper case (state 3) letters represent significant differences among groups (*p* < 0.05, ANOVA, Tukey’s post-hoc analysis).

The transfer and subsequent acclimation of rainbow trout to seawater did not significantly affect resting 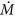
_O_2__ with values ranging between ~55 and 62 mg O_2_ kg^−1^ h^−1^ (ANCOVA F_7,55_ = 0.480, *p* = 0.845, Fig. 2A). A significant effect in state 2′ intestinal mitochondrial respiration rate (*i.e.* leak respiration rate) was detected following a transfer and/or subsequent acclimation to seawater (ANOVA F_7,49_ = 7.087, *p* < 0.001, Fig. 2B). Figure 2B shows that although state 2′ mitochondrial respiration rates were significantly elevated on day 2 (2.14 ± 0.06 pmol s^−1^ mg^−1^, *p* = 0.009), values tended to be slightly lower when compared to freshwater (1.37 ± .37a pmol s^−1^ mg^−1^) from day 4 onwards (1.04 ± 0.07 pmol s^−1^ mg^−1^). Seawater transfer and subsequent acclimation did not significantly affect state 3 intestinal mitochondrial respiration rates (*i.e.* OXPHOS respiration rate) (ANOVA F_7,49_ = 1.223, *p* = 0.308, Fig. 2B). Figure 2B shows that, although not significant, values tended to be slightly higher when compared to freshwater (3.64 ± 0.23 pmol s^−1^ mg^−1^) from day 4 onwards (4.14 ± 0.21 pmol s^−1^ mg^−1^).

**Figure 3:**
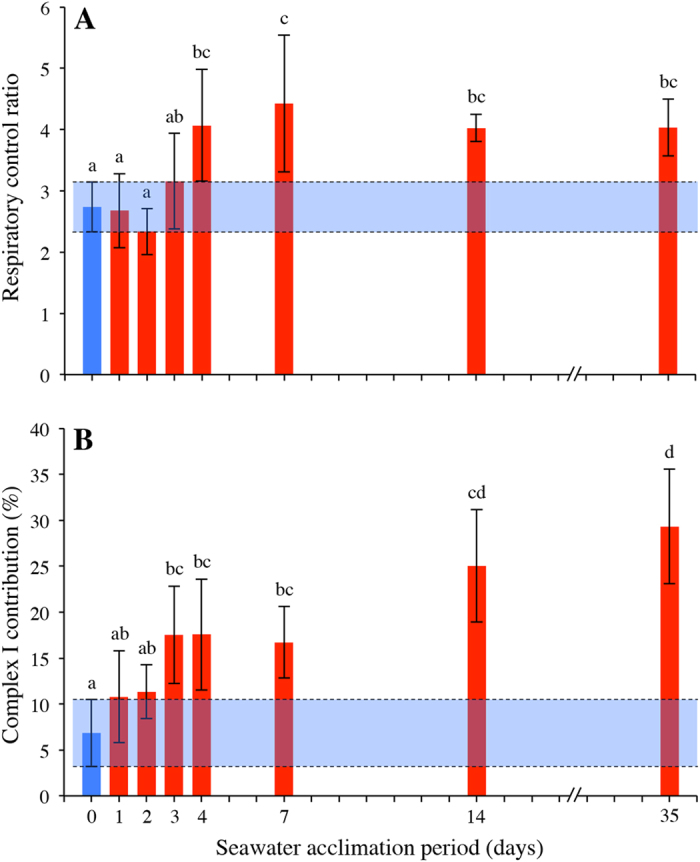
Mitochondrial coupling and the contribution to electron transport of complex I of rainbow trout in freshwater and seawater. (**A**) mitochondrial coupling (RCR) and (**B**) contribution to electron transport of complex I of rainbow trout in freshwater (blue bar) and during the transfer and/or subsequent acclimation to seawater (red bars) at 10.0 ± 0.1 °C (n = 7 individual trout for each time point except day 3 and 35 where n = 8). Variables are reported as means and the error bars represent the upper and lower bands of the 95% CI. for each mean (horizontal blue shaded rectangles represents 95% CI for freshwater acclimated trout). Dissimilar letters represent significant differences among groups (*p* < 0.05, ANOVA, Tukey’s post-hoc analysis).

Although mitochondrial respiration rates (*i.e.* state 3 and 2′) and 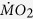 of rainbow trout did not greatly change after transfer to seawater, RCR was significantly affected (ANOVA F_7,49_ = 8.029, *p* < 0.001, Fig. 3A) due to the slight modifications in state 2′ and state 3, as described above. RCR was higher after 4 days of seawater acclimation when compared to freshwater values (*p* = 0.031) and remained elevated for the remainder of the acclimation period (*p* = 0.002, 0.041, 0.039 for comparisons between freshwater and day 7, 14, 35, respectively; Fig. 3A). RCR was also positively correlated with intestinal NKA activity (r_(55)_ = 0.63, *p* < 0.001). The relative contribution to electron transport of complex I was also significantly affected (ANOVA F_7,49_ = 12.722, *p* < 0.001, Fig. 3B) as it increased ~5-fold during the 35 day acclimation period (*p* = 0.011, 0.015, 0.033, <0.001, <0.001 for comparisons between freshwater and day 3, 4, 7, 14, 35, respectively), which translated into an increased RCR. Consequently, the temporal dynamics of the relative contribution to electron transport of complex II during seawater acclimation was a mirror image (*i.e.* direct opposite) of that displayed by complex I (*i.e.* significantly decreased from freshwater level after 3 days of seawater acclimation to reach levels ~5-fold lower).

**Figure 4:**
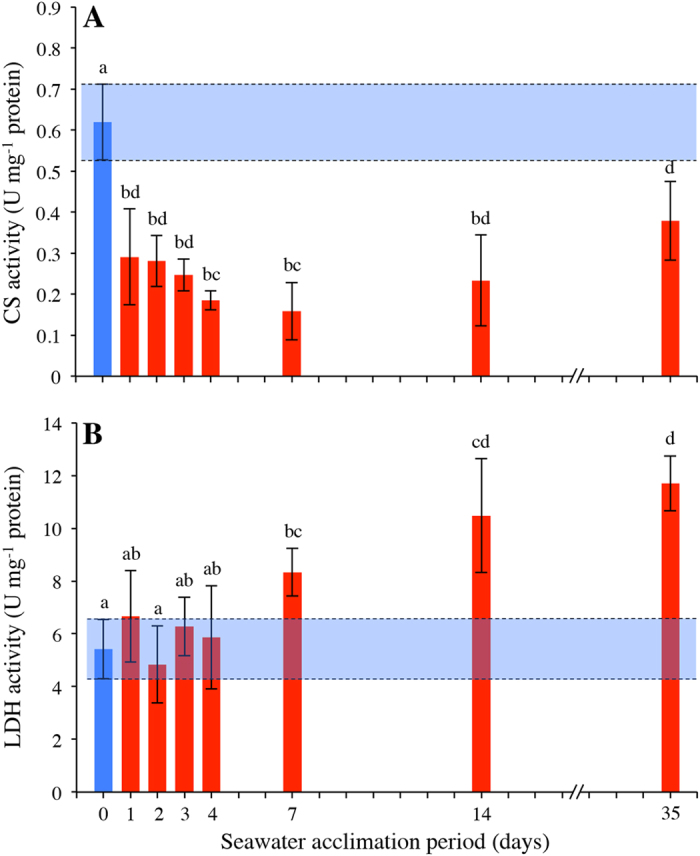
Activity of citrate synthase and lactate dehydrogenase in the intestine of rainbow trout in freshwater and seawater. (**A**) citrate synthase (CS) and (**B**) lactate dehydrogenase (LDH) activity in the intestine of rainbow trout in freshwater (blue bar) and during the transfer and/or subsequent acclimation to seawater (red bars) at 10.0 ± 0.1 °C (n = 8 individual trout for each time point). Variables are reported as means and the error bars represent the upper and lower bands of the 95% CI for each mean (horizontal blue shaded rectangles represents 95% CI for freshwater acclimated trout). Dissimilar letters represent significant differences among groups (*p* < 0.05, ANOVA, Tukey’s post-hoc analysis).

Intestinal CS and LDH activity were both significantly affected by the transfer and/or subsequent acclimation to seawater (CS: ANOVA F_7,56_ = 17.503, *p* < 0.001, Fig. 4A and LDH: ANOVA F_7,56_ = 15.406, *p* < 0.001, Fig. 4B). Following a transfer to seawater, intestinal CS activity decreased to reach levels ~4-fold lower than activity in freshwater (*p* < 0.001 for comparisons between freshwater and day 1–7). Although CS activity began to increase after 7 days, the level of CS activity had not yet returned to freshwater levels after 35 days of seawater acclimation (*p* < 0.001). In contrast to CS activity, intestinal LDH activity was higher after 7 days of seawater acclimation when compared to activity in freshwater and reached levels >2-fold higher than that observed in freshwater at the end of the acclimation period (*p* = 0.040, <0.001, <0001 for comparisons between freshwater and day 7, 14, 35, respectively). Intestinal LDH activity was also positively correlated with intestinal NKA activity (r_(64)_ = 0.46, *p* < 0.001).

## Discussion

Although anadromous salmonids such as rainbow trout are able to tolerate short-term osmotic and ionic perturbations that accompany a transition to seawater, hypo-osmoregulatory mechanisms must be initiated relatively quickly as prolonged disruption of osmotic homeostasis ultimately results in death^4^. In the present study, rainbow trout experienced significantly increased plasma osmolality and ion concentrations for four days following a transfer to seawater, after which osmotic homeostasis was regained. The time-course for the abovementioned perturbation in osmotic homeostasis is similar to findings from other studies on anadromous salmonids, which indicate that the return of water and ionic balance (~5 days post-transfer) coincided with increased branchial and pyloric caeca NKA activity^14^,^15^. Indeed, elevated gill and intestinal NKA activity is thought to be crucial for survival in seawater as it provides the driving force for the trans- and paracellular transport of monovalent ions^26^. Although elevated gill and intestinal NKA activity has been demonstrated in a wide range of anadromous salmonids migrating to seawater (see ref. 4 and references within), as far as we are aware the time course of changes in intestinal NKA activity in response to salinity changes had not previously been documented. Similar to the findings from the gills and pyloric caeca^15^,^27^, increased intestinal NKA activity of rainbow trout in the present study also coincided with the return of osmotic homeostasis indicating that NKA activity is simultaneously up-regulated in the gills, pyloric caeca and intestine following seawater transfer. Interestingly, the time course for the up-regulation in branchial and intestinal NKA activity of rainbow trout closely matches the circulatory changes (*i.e.* elevated cardiac output and gastrointestinal blood flow) that occur in this species during seawater transfer^9^,^16^,^17^. This would allow the fish to restore hydromineral balance by transporting absorbed ions from the intestine to the gills for excretion^28^.

The abovementioned elevations of NKA activity in osmoregulatory organs should incur an increased ATP demand, and thus one could expect an increased metabolic cost for anadromous salmonids osmoregulating in seawater. Yet at the whole animal level, no significant differences were observed in resting 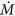
_*O*_2__ of rainbow trout in freshwater or seawater, which may have positive implications for the relative fitness of a migrating individual, as they may be able to maintain their aerobic capacity for locomotion (*i.e.* foraging and predator avoidance) and growth when at sea^2^,^3^. The lack of a significant change in resting 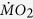 of rainbow trout during the transfer and subsequent acclimation to seawater may indicate that: (i) 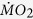 measures may not be sensitive enough to detect the potentially minor changes in osmoregulatory costs and thus investigations at the organ and sub-cellular level are required^24^,^29^, (ii) mitochondrial respiration and thus ATP production by the mitochondria that are located in essential osmoregulatory organs such as the intestine varies between the different environments^19^, or (iii) that ATP demand is supplied via an increased anaerobic capacity. *In vivo* analysis of tissue energetics in intact cells would be the most physiologically relevant approach to investigate these possibilities. However, the scope for *in vivo* measurements of mitochondrial respiration is limited, as the main mitochondrial effectors cannot penetrate the cellular membrane^30^. Even though the dynamics of a range of processes that are under the control of cytoplasmic proteins and signals are lost in permeabilized tissues, the basic physical interactions will remain intact (see ref. 30 and references within). Therefore, an *in situ* approach (*i.e.* permeabilized tissue) provides us essential information concerning the effects that salinity acclimation may have on mitochondrial function or enzymatic activity in the intestine of a hypo-osmoregulating salmonid.

Intestinal mitochondrial respiration rates (*i.e.* leak respiration, state 2′ and OXPHOS respiration, state 3) and indices for mitochondrial coupling (*i.e.* RCR) reported in the present study are lower (state 2′: ~4-fold lower, state 3: ~10-fold lower, RCR: ~2.5-fold lower) than those observed in another study measuring mitochondrial function in permeabilized cardiac tissue of freshwater-acclimated rainbow trout at 10 °C, which is not surprising considering that the heart is a highly oxidative tissue with a substantial amount of mitochondria^31^. However, our results are within the same range as those reported in a recent study performed on permeabilized hearts and brains of killifish (*Fundulus heteroclitus*) measured at 5 and 15 °C^32^. Direct comparisons of indices for mitochondrial function between the present study and others are however difficult and should be treated with caution due to the inherent differences that exist in factors such as experimental protocols, fish species and acclimation temperatures. With respect to the effect that the transfer and subsequent acclimation to seawater had on energy metabolism at the sub-cellular level of rainbow trout, the present study reveals that similar to resting 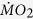, intestinal mitochondrial respiration rates (*i.e.* state 2′ or state 3) of rainbow trout did not greatly differ. Instead, mitochondrial coupling (*i.e.* RCR) and LDH activity increased during seawater acclimation. As RCR encapsulates the main function of mitochondria (*i.e.* the ability to idle at a low rate whilst being able to respond to ADP by producing ATP at a high rate^33^), a significantly elevated RCR might indicate that the coupling between electron transport (and hence oxygen consumption) and the phosphorylation of ADP has improved, especially when one considers that state 2′ does not display major modifications (with the exception of day 2) and hence that seawater acclimation does not result in unsustainable oxygen consumption. Furthermore, the increase in RCR and LDH activity were strongly positively correlated with NKA activity, indicating that they may constitute important underlying mechanisms for the supply of ATP required for the active transport of ions in the intestine. These findings highlight the importance of combining sub-cellular and whole animal measurements of metabolism when investigating the relationship between energy metabolism and life-history strategies, since the rate of ATP generation is dependent on both the rate of oxygen consumption and mitochondrial efficiency^19^. Indeed, variations in mitochondrial coupling and anaerobic capacity may be underlying factors contributing towards the highly conflicting results previously reported in the literature concerning the effect of water salinity on energy metabolism of euryhaline teleosts^8^.

An increased RCR indicates that the mitochondria possess an increased capacity for substrate oxidation and ATP turnover, whilst reducing proton leak across the inner mitochondrial membrane^33^. The lack of significant reductions in proton leak (state 2′) or increases in the capacity for substrate oxidation (state 3) suggests that the increased RCR during seawater acclimation is most likely due to slight modulations of these parameters (*i.e.* slight reductions in state 2′ coinciding with slight elevations in state 3), which allow for a greater capacity to pump protons from the matrix to the inter-membrane space by the mitochondrial complexes. This increased capacity would consequently result in an increased amount of ADP phosphorylated to ATP by the protein complex ATP synthase^34^. The significant increase in mitochondrial coupling coincided with an increase in the relative contribution to electron transport of complex I concomitant with a decrease in the relative contribution of complex II. Interestingly, the same pattern (an increase and decrease in the relative contributions of complexes I and II, respectively) has recently been observed after an acute temperature increase in the heart of cold-acclimated *Fundulus heteroclitus*^35^. Combined with our observations, this suggests that a shift in the relative contributions of complexes I and II might be an important factor during a change in environmental conditions. If proton leak is not significantly increased (as indicated by our results), the relative increased capacity for electron flow via complex I instead of complex II might theoretically result in an increased capacity for pumping protons and ATP turnover^36^. The relative increase in the electron flow through complex I would require an increased capacity for NADH production via pyruvate oxidation and the tricarboxylic acid cycle^34^. However, the present study demonstrates that CS activity in the intestine was significantly reduced upon exposure to seawater while LDH was increased, which suggests that a higher proportion of pyruvate produced via glycolysis was diverted towards the production of lactate^34^.

CS activity is often regarded as a good marker for mitochondrial content^37^, and it is therefore possible that our results reflect a reduction in the amount of mitochondria during the acclimation to seawater. However, if this was the case, then we should have observed parallel changes in both state 2′ and state 3. Since CS is regarded as a pacemaker enzyme in the tricarboxylic acid cycle^38^, a reduction in CS activity may also reflect a reduction in the capacity to produce the reducing equivalents NADH (necessary electron donor for complex I) and FADH_2_ (necessary electron donor for complex II). The decreased capacity for FADH_2_ production by the tricarboxylic acid cycle can explain the decreased contribution to electron transport of complex II, whereas the reduced capacity for NADH production through this pathway might indicate that this reducing agent is supplied via other sub-cellular pathways.

If lipid stores were used to sustain the provision of NADH to complex I, one would expect an increase in CS activity as fatty acid oxidation results in increased acetylcoenzyme A production, which is the main substrate for CS^34^. Interestingly, research on the intermediary metabolism of anadramous Arctic char (*Salvelinus alpinus*) revealed that during seawater acclimation there were significant increases in amino acid metabolism in all tissues investigated (*i.e.* gill, liver, white and red muscle), indicating that these fish have an enhanced capacity for energy production from amino acids^27^. Therefore, the most parsimonious explanation for our results is that the catabolism of amino acids during the initial 35 days of seawater acclimation allow an increased capacity for NADH production in the mitochondria through anaplerotic reactions that provide tricarboxylic acid intermediaries (although not at the level of complex II), which results in an increased mitochondrial capacity via the electron supply to complex I. Indeed, it has been previously demonstrated that after 96 h in seawater, the enzymatic activity of aspartate aminotransferase in the gills of Arctic char was significantly elevated, which suggests that the transdeamination of amino acids such as aspartate might be an important pathway for ATP production^27^. This study also reports a significant increase in the NKA activity of gills after 96 h in seawater, similar to what we observed in the intestine. However, in contrast with our results, an increase in CS activity was observed in the gills of Arctic char after 96 h^27^, which indicates that different osmoregulatory organs may have different mechanisms for coping with the increased ATP demand by the NKA. Interestingly, during the latter stages of seawater acclimation it seemed that intestinal CS activity was gradually returning to levels observed in freshwater. The combination of increased intestinal CS activity (*i.e.* increased capacity for NADH production via pyruvate oxidation and the tricarboxylic acid cycle) and catabolism of amino acids may explain why the relative contribution to electron transport of complex I continues to increase over time. However, the long term response (>35 days of seawater acclimation) of intestinal CS activity remains unknown and should be evaluated to confirm this hypothesis.

Interestingly, increased mitochondrial function has been implicated to affect performance in a range of whole-organism traits such as growth, exercise capacity and reproductive output (see ref. 19 and references within). Thus, in addition to minimizing the aerobic cost of osmoregulation in seawater, increased mitochondrial coupling may potentially be an important factor contributing towards the faster growth and larger body size of anadromous individuals that have undertaken the migration to sea when compared to non-migratory conspecifics^1^,^3^. The increase in mitochondrial coupling of rainbow trout in seawater compared to freshwater may also represent a life-history trade off, as mitochondria are the main producers of reactive oxygen species (ROS) and mitochondrial coupling is positively related with the production of ROS^19^. Enhanced levels of ROS have the potential to induce damage to cellular macromolecules through oxidative stress, which has been proposed as an important factor underlying cellular and whole-organism senescence^39^. Indeed, a study on anadromous sturgeon (*Acipenser naccarii*) demonstrated that osmoregulation in seawater was associated with an increased level of oxidative stress causing major physiological changes in the fish^40^. Therefore, when at sea, natural selection may favour phenotypes with relatively high mitochondrial coupling (*i.e.* leads to faster growth, increased body size, minimizes aerobic costs of osmoregulation), whereas in freshwater environments a lower mitochondrial coupling and reduced oxidative stress may become the priority^19^. This hypothesis is in accordance with Blier *et al*.^41^ who suggested that the vital functions of mitochondria (*i.e.* producing an adequate supply of ATP and regulating ROS production) clearly pinpoint this organelle as a key target for natural selection. The capacity of anadromous fish to modulate their mitochondrial metabolism to maintain their osmotic balance could therefore have been a major force driving adaptation to different aquatic environments and warrants further investigation.

By integrating whole animal and sub-cellular metabolic measurements, this study presents novel and significant findings that demonstrate how an anadromous salmonid, such as rainbow trout, is able to transform from a hyper- to hypo-osmoregulatory state without incurring significant increases in resting 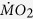. This may have positive implications for the relative fitness of the migrating individual, as aerobic capacity may be maintained for other vital activities such as locomotion and growth. Further investigation into the underlying metabolic mechanisms potentially fueling the osmoregulatory machinery in the intestine suggests that rather than quantitatively increasing mitochondrial oxygen consumption to satisfy energetic demands in seawater, mitochondrial coupling increased in combination with potential increases in anaerobic capacity. The ability to modulate mitochondrial metabolism in order to maintain osmotic balance suggests that mitochondria of anadromous fish may have been a key target for natural selection, driving species adaptations to different aquatic environments. Furthermore, the increased contribution of complex I along with the increased mitochondrial coupling suggests that NAD-linked substrates are preferentially used in the intestine of rainbow trout in seawater instead of the FAD-linked substrates that are preferred in freshwater. As mitochondrial coupling is associated with ROS production, the variations in coupling may also represent a life-history trade off for rainbow trout residing in freshwater and seawater environments.

## Methods

### Experimental animals and holding conditions

Rainbow trout (*Oncorhynchus mykiss*, (Walbaum, 1792), body mass:~300 g, mixed sex, n: ~120) were obtained from a local hatchery (Antens Laxodling AB, Alingsås, Sweden) and held at 10 °C in 500 L rectangular tanks (~60 individuals per tank) containing recirculating, aerated freshwater (salinity 0.1 ppt; pH 7.8; conductivity 255 μS cm^−1^; [Na^+^] 5, [K^+^] 0.2, [Ca^2+^] 0.6 mmol L^−1^). The animals were held under these conditions on a 12:12 h light:dark photoperiod for at least 6 weeks prior to experimentation and fed once a week *ad libitum* with dry commercial trout pellets (Protec Trout pellets, Skretting, Stavanger, Norway). Seawater acclimation was accomplished by netting and directly transferring all the fish, except ~30 individuals (remained in one freshwater tank), into identical 500 L rectangular tanks (~30 individuals per tank) containing recirculating, aerated artificial seawater (salinity 30 ppt; pH 7.6–8.0; conductivity 47 mS cm^−1^; [Na^+^] 420, [K^+^] 5, [Ca^2+^] 7 mmol L^−1^; source of artificial sea salt: Grotech, GmbH, Ahorn, Germany) at 10 °C. Fish acclimating to seawater were fed the same rations as in freshwater. Mortality during seawater acclimation was <5% (*i.e.* 5 individuals). Animal care and all physiological experimental procedures were performed in accordance with guidelines and regulations approved by the regional ethical committee of Gothenburg in Sweden (ethical permit 167-2013).

### Experimental protocol

To prevent the confounding effects of postprandial processes on whole-animal and sub-cellular metabolism^42^,^43^, fish were fasted for 5–7 days prior to measurements to ensure the complete evacuation of food contents from the gastrointestinal tract (see ref. 44 and references within). Data were obtained from freshwater-acclimated rainbow trout (day 0, n = 8) and at 7 stages of seawater acclimation (*i.e.* 1, 2, 3, 4, 7, 14 and 35 days after transfer to seawater, n = 8 individuals for each time period). Since fasting has been documented to influence whole-animal and sub-cellular metabolism (*i.e.* resting 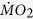 and mitochondrial coupling^19^,^45^,^46^,^47^), a fasting period as similar as possible was used for each acclimation group although due to the limited number of seawater acclimation tanks there were slight differences in the fasting duration (*i.e.* 6 days of fasting for all acclimation groups except SW day 1 and day 3, which had fasting periods of 5 and 7 days, respectively). Due to the high number of fish in the tanks, it was possible to randomly net 8 individuals from a tank in 1–2 sweeps with a large pole net. Body mass of sampled fish (n = 64, mass = 299 ± 8 g) did not significantly differ between the different acclimation groups (ANOVA F_7,56_ = 1.235, *p* = 0.299).

### Measurements of resting 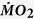


Resting 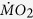 was determined using best practices for intermittent-flow respirometry^48^. Fish were individually placed into one of eight identical custom-made Perspex respirometers (volume = 3 L), which were submerged in a larger experimental tank (volume = 500 L) with recirculating aerated freshwater or seawater at 10.0 ± 0.1 °C. The partial pressure of oxygen in the water within each respirometer was measured continuously at 1 Hz using a FireSting O_2_ system (PyroScience, Aachen, Germany). The signal was relayed to a PowerLab 8/30 system (ADInstruments, Castle Hill, Australia) and the data were collected on a PC using ADInstruments acquisition software Chart^TM^ 7 Pro v7.2.5. Automated flush pumps refreshed the water in the respirometers for 12 min in every 15 min period. The slope of the decline in the partial pressure of oxygen in the water within the respirometers between flush cycles (*i.e.* when the respirometer was closed) was then used to calculate 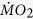 using the following formula:


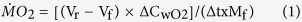


where V_r_ is the volume of the respirometer, V_f_ is the volume of the fish (assuming that the overall density of the fish is 1 g per mL of tissue, thus V_f_ = mass of the fish, M_f_), ∆C_wO2_ is the change in the oxygen concentration of the water within the respirometer (C_wO2_ is the product of the partial pressure and capacitance of oxygen in the water, the latter being dependent on salinity and temperature) and ∆t is the time during which 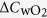 is measured. The fish were left undisturbed in the respirometers for 24 h while 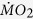 was measured. Resting 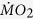 for each individual was determined as the mean of the lowest 10% of oxygen consumption rate measurements recorded during the 24 h period. Outliers, which were considered to be > 2 standard deviations below the mean of the lowest 10% of values, were excluded from the analysis. After 24 h, fish were removed from the respirometers and euthanized with a sharp cranial blow.

### Dissection and sample preparations

Body mass (g) of each euthanized individual was determined prior to removing a blood sample by caudal puncture with a heparinized syringe, which was subsequently placed on ice until further processing and analysis (see *Determination of osmotic balance*).

Following the removal of a blood sample, a ventral incision was made and the proximal intestine was carefully dissected out of the fish. For the mitochondrial respiration analysis, a section of the proximal intestine (~1 cm) between the pyloric caeca and ileorectal valve was placed into a petri dish containing a modified BIOPS relaxing solution (2.77 mM CaK_2_EGTA, 7.23 mM K_2_EGTA, 5.77 mM Na_2_ATP, 6.56 mM MgCl_2_, 20 mM taurine, 15 mM Na_2_phosphocreatine, 20 mM imidazole, 50 mM MES, 2 μg mL^−1^ aprotinin, pH 7.1^49^) and immediately placed on ice until further processing and analysis (see *Mitochondrial respiration analysis*). For the enzymatic assays, another section of the proximal intestine (~1 cm) was opened longitudinally with the luminal side facing upwards. The luminal side was gently rinsed with intestinal SEI buffer (200 mM glycine, 45 mM EDTA, 50 mM EGTA, 300 mM sucrose, 50 mM imidazole, 2 μg mL^−1^ aprotinin, 1 mM PMSF, pH 7.6^50^) prior to scraping off the mucosa with a glass microscope slide. The mucosal sample was then weighed and transferred to an eppendorf tube containing intestinal SEI buffer (volume of SEI buffer = 10 x wet weight of mucosal sample), which was immediately placed on ice until further processing and analysis (see *Enzymatic assays*). Collection of the abovementioned tissue samples was performed on ice.

### Determination of osmotic balance

The blood samples were spun at 5000 g for 5 min in a Jouan A14 centrifuge (Thermo Fisher Scientific, MA, USA). The plasma was removed and immediately placed on ice. Plasma osmolality was determined using an Advanced^®^ Model 3300 micro-osmometer (Advanced Instruments, MA, USA). Plasma [Na^+^], [K^+^] and [Ca^2+^] were determined with an Eppendorf Elex 6361 flame photometer (Eppendorf, Hamburg, Germany), zeroed with a Lithium diluent blank and calibrated with a flame photometer serum standard (Eppendorf, Hamburg, Germany), using plasma diluted in 5 mM Lithium diluent (1:50).

### Mitochondrial respiration analysis

Intestinal tissue permeabilizations were conducted at 4 °C with sections of the proximal intestine sampled from trout. Tissues were initially mechanically permeabilized in a petri dish containing modified BIOPS with two pairs of sharp forceps to achieve a thin mesh of tissue^30^. The tissues were then transferred to modified BIOPS complemented with 50 μg mL^−1^ saponin and gently mixed at 4 °C for 30 min. Tissues were then rinsed at 4 °C for 10 min in respiration medium (140 mM KCl, 5 mM KH_2_PO_4_, 20 mM HEPES, 3 mM MgCl_2_, 1% BSA wt/vol, pH 7.2). Mitochondrial respiration was then measured using a respirometry setup previously described^31^. Briefly, duplicates of permeabilized intestinal tissue (blotted dry weight =21.6 ± 0.3 mg) from each individual fish were transferred to individual tube-shaped glass respirometry chambers (vertical mini chambers, LoligoSystems, Tjele, Denmark) filled with air-saturated respiration medium and maintained at 10.0 ± 0.1 °C. The volumes of the chambers were carefully determined (649.1, 630.0, 588.1 and 525.3 μL) and the concentration of substrates used in the experiments was adjusted in accordance. Constant stirring of the medium and the sample inside the chamber was accomplished using a Control unit for mini stirrer (LoligoSystems, Tjele, Denmark). A total of four chambers were used, which allowed the simultaneous recording of oxygen consumption from duplicates of the proximal intestine of two fish. A small section of O_2_ sensor spot material (~2 mm^2^, LoligoSystems, Tjele, Denmark) was attached to the wall inside each respirometer and the partial pressure of oxygen of the respiration medium was measured at 1 Hz by focusing the fibre-optic (*i.e.* optodes) on the sensor spot from outside the respirometer and relaying the signal to the FireSting O_2_ system (PyroScience, Aachen, Germany). The signal was relayed to a PowerLab 8/30 system (ADInstruments, Castle Hill, Australia) and the data were collected on a PC using ADInstruments acquisition software Chart^TM^ 7 Pro v7.2.5. The optodes were calibrated with air-saturated respiration medium at 10.0 ± 0.1 °C and zero-O_2_ measurements were taken after the addition of sodium dithionite.

Using this setup, we determined a range of indices for mitochondrial function including state 2′ and state 3 mitochondrial respiration rates (when electrons are supplied simultaneously to complexes I and II, and converge at the ubiquinone junction)^51^, the respiratory control ratio (RCR), and the relative contributions of complex I and II. State 2′ is a measure of the leak respiration rate and is defined as the controlled respiration prior to the addition of ADP^51^. State 2′ was achieved via the addition of substrates that stimulate NADH production (5 mM pyruvate, 0.5 mM malate and 10 mM glutamate) and FADH_2_ production (10 mM succinate) to the respiration medium in the chambers containing the permeabilized tissues (all concentrations and substrates were optimized to yield the highest oxygen consumption). State 2′ was used in this study as it has been suggested to be functionally the same as state 4^52^, and also due to the fact that ATP is rapidly cycled back to ADP in permeabilized tissues, which prevents a proper assessment of state 4^53^. Following the determination of state 2′, 5 mM ADP was added to the chambers to achieve state 3 respiration for complexes I and II (OXPHOS respiration rate). From these measures, the RCR was calculated (see equation 2 below)^30^. RCR, a traditional approximation of mitochondrial coupling, has previously been demonstrated to provide a reasonable measure of the coupling between the electron transport system and the oxidative phosphorylation system^30^,^53^,^54^,^55^,^56^,^57^,^58^. Following the determination of state 3, 10 μM cytochrome *c* from equine heart was added to control for the functional integrity of the outer mitochondrial membrane (samples were discarded if an increase of more than 10% of oxygen consumption was detected after cytochrome c addition due to potential damage to the outer mitochondrial membrane). Then 0.5 μM rotenone, an inhibitor of complex I, was added to the chambers and the relative contribution to electron transport of complex I was calculated (see equation 3 below)^35^,^58^. Finally, antimycin A (2.5 μM, inhibitor of complex III) were added to the chambers and the residual oxygen consumption (oxygen consumption after the addition of antimycin A) was subtracted from the other respiration rates. Duplicates of the mitochondrial respiration rates were averaged for each individual and expressed as mean respiration rates in picomoles of O_2_ consumed per second per milligram of tissue (pM O_2_ s^−1^ mg^−1^).


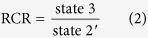



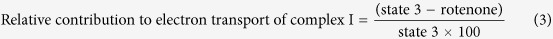


### Enzymatic assays (Na^+^/K^+^-ATPase activity, LDH and CS)

Mucosal samples from each fish were homogenized by 20 strokes with a glass/glass tissue grinder (Contes glass, Vineland, NJ, USA) on ice and then centrifuged at 3000 g for 3 min at 4 °C in an Eppendorf centrifuge 5415 R (Eppendorf, Hamburg, Germany)^50^. The supernatant (*i.e.* mucosal homogenate) was saved, immediately placed on ice and then used for activity assessment.

Intestinal Na^+^/K^+^-ATPase activity was determined according to the method described by McCormick^59^. The mucosal homogenate (10 μL) was incubated in duplicate with 150 μL assay medium (4 units LDH mL^−1^, 5 units PK mL^−1^, 2.8 mM PEP, 0.7 mM ATP, 0.22 mM NADH, 50 mM imidazole, pH 7.5) and 50 μL salt solution (50 mM imidazole, 189 mM NaCl, 10.5 mM MgCl_2_.6H_2_O, 42 mM KCl, pH 7.5), with and without 0.5 mM ouabain in a 96-well microplate. As the ouabain-sensitive hydrolysis of ATP is enzymatically coupled to the oxidation of NADH (reduced form), the linear rate of NADH disappearance was measured at 340 nm (SpectraMax 190, Molecular Devices Corp., Menlo Park, CA, USA) for 10 min at 25 °C. Specific Na^+^/K^+^-ATPase activity was determined by comparing differences in ATP hydrolysis in the absence and presence of ouabain.

LDH and CS activity were determined according to the method described by Ekström *et al*.^60^. The mucosal homogenate used for the determination of LDH and CS activity was initially diluted ten-fold. For the LDH assay, the diluted homogenate (5 μL) was then incubated with 200 μL reaction medium (100 mM KH_2_PO_4_, 0.16 mM NADH, 0.4 mM pyruvate, pH 7.0) and read in triplicate at 340 nm for 4 min at 25 °C (ε_NADH_ = 6.22 mL cm^−1^ μmol^−1^). For the CS assay, the diluted homogenate (5 μL) was incubated with 250 μL reaction medium (100 mM imidazole, 0.1 mM DTNB, 0.1 mM AcetylCoA, 0.15 mM oxaloacetic acid, pH 8.0) and read in triplicate at 412 nm for 4 min at 25 °C (ε_DTNB_ = 13.6 mL cm^−1^ μmol^−1^).

Protein concentrations of all samples were determined using a DC Protein Assay (Bio-Rad, CA, USA) using BSA as a standard. All enzyme activities were expressed as U mg^−1^ protein, where U represents 1 μmol of substrate transformed to product per minute.

### Statistical analyses

The sample sizes for each variable were chosen based on previous studies in our laboratory, as well as from a pilot study carried out prior to the experiments. Statistical analyses were performed using SPSS Statistics 21 (IBM Corp., Armonk, NY, USA). All data subjected to statistical analyses were assessed for outliers, normality (Shapiro-Wilk’s test, *P* > 0.05) and homogeneity of variance (Levene’s test, *P* > 0.05). Relationships between body mass and the measured traits were assessed by visual inspection of a scatterplot. If a relationship existed between body mass and a measured trait (*i.e.* resting 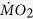), then a one-way ANCOVA was used to determine whether there were any statistically significant differences between the means of the dependent variable during seawater acclimation using body mass as a covariate. To meet the assumptions of the one-way ANCOVA, a natural logarithmic transformation was applied to body mass. Furthermore, we checked to ensure that there were no significant interaction terms between the dependent variable and covariate (Interactions: resting 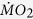 *vs.* body mass F_7,48_ = 1.012, *P* = 0.435). If no relationship existed between body mass and the measured traits (*i.e.* plasma osmolality, NKA activity, state 2′, state 3, RCR, complex I, CS and LDH activity), then a one-way ANOVA with a Tukey’s post hoc test was used to determine whether there were any statistically significant differences between the means of the dependent variables during seawater acclimation. For the dependent variables that violated the assumption of homogeneity of variance, we instead used Welch’s ANOVA with a Games-Howell post hoc test. To determine the strength and direction of relationships between specific variables (*i.e.* plasma osmolality *vs.* ion concentrations, RCR *vs.* NKA activity and LDH *vs.* NKA activity), a Pearson product-moment correlation was used to generate correlation coefficients (r). F- and P- values obtained from the statistical analyses are reported throughout the text, with significance defined at *p* < 0.05. Unless otherwise specified, all data are presented as means ± S.E.M.

## Additional Information

**How to cite this article**: Brijs, J. *et al*. Increased mitochondrial coupling and anaerobic capacity minimizes aerobic costs of trout in the sea. *Sci. Rep.* **7**, 45778; doi: 10.1038/srep45778 (2017).

**Publisher's note:** Springer Nature remains neutral with regard to jurisdictional claims in published maps and institutional affiliations.
